# Use of Anti-Granulocyte Scintigraphy with 99mTc-Labeled Monoclonal Antibodies for the Diagnosis of Periprosthetic Infection in Patients after Total Joint Arthroplasty: A Diagnostic Meta-Analysis

**DOI:** 10.1371/journal.pone.0069857

**Published:** 2013-07-26

**Authors:** Dan Xing, XinLong Ma, JianXiong Ma, Jie Wang, Yang Chen, Yang Yang

**Affiliations:** 1 Department of Orthopaedics Institute, Tianjin Hospital, Tianjin, China; 2 Department of Orthopaedics, Tianjin Medical University General Hospital, Tianjin, China; 3 Department of Orthopaedics, Tianjin Gongan Hospital, Tianjin, China; University of Leicester, United Kingdom

## Abstract

The accurate diagnosis of periprosthetic joint infections (PJI) is crucial for therapy and the prevention of complications. No diagnostic test of PJI is 100% accurate. The aim of this study was to assess the use of anti-granulocyte scintigraphy using 99 mTc-labeled monoclonal antibodies to diagnose PJI after total joint arthroplasty. A systematic search of all relevant studies published through January 2013 was conducted using the MEDLINE, EMBASE, OVID, and ScienceDirect databases. Observational studies that assessed the accuracy of the anti-granulocyte scintigraphy with monoclonal antibodies or antibody fragments labeled with technetium 99 m in diagnosis for PJI and provided data on specificity and sensitivity were identified. Standard methods recommended for meta-analysis of diagnostic accuracy were used. Nineteen studies were eligible for inclusion. The results demonstrated that the area under the summary receiver operator curve was 0.88, and the diagnostic accuracy (Q*) was 0.81. Additionally, the diagnostic odds ratio (DOR) was 18.76 with a corresponding 95% confidence interval of 10.45–33.68. The pooled sensitivity and specificity of the diagnostic method for the diagnosis of PJI were 83% and 79%, respectively, while the pooled positive likelihood ratio (PLR) was 3.56, and the negative likelihood ratio (NLR) was 0.26. Anti-granulocyte scintigraphy using 99 mTc-labeled monoclonal antibodies has a reasonable role in the diagnosis of PJI after total joint arthroplasty. Due to the limitations of the present meta-analysis, additional high-quality original studies are required to confirm the predictive value.

## Introduction

Joint loosening, heterotopic ossification, periprosthetic fractures, luxation, osteolysis and periprosthetic joint infections (PJI) are the failures of joint arthroplasty or complications following joint arthroplasty. In particular, PJI occur in 1–2% of the primary implants and in 3–5% of revision implants [1], [2], and PJI may be the most devastating complication of total joint arthroplasty. Despite recent advances in prophylaxis, the prevalence of PJI is increasing [3]. Therefore, the accurate diagnosis of PJI is crucial for therapy and the prevention of complications. A failure to recognize PJI may lead to the unintended implantation of a new prosthesis into an infected surgical site. Without the appropriate debridement of the joint or antibiotic treatment, this implantation may result in persistence of the infection and early failure of the revision surgery. Conversely, an erroneous diagnosis of PJI in the absence of infection may result in unnecessary surgical procedures and inappropriate treatment with a prolonged course of parenteral antibiotics.

The diagnosis of PJI poses numerous challenges. Differentiating PJI from aseptic loosening is very difficult because these conditions may present with similar clinical and histopathological signs. No diagnostic test of PJI is 100% accurate. The diagnosis of PJI is more challenging when clinical signs are subtle or absent [4]. Furthermore, various modalities, including clinical signs, hematology, bacteriological culture, and radiographs, are unreliable or have controversial efficacy [5]. Infections are diagnosed primarily on the basis of laboratory tests measuring C-reactive protein (CRP), the erythrocyte sedimentation rate (ESR), the peripheral leukocyte count, histological examination and cell cultures, as well as cell counts in the infected area [6]. However, such serum markers can be affected by conditions remote from the joint of interest. Moreover, diagnosis of PJI is frequently supported by anatomical imaging. However, anatomical imaging methods such as plain X-ray, computed tomography (CT) and magnetic resonance imaging (MRI) have lower sensitivity in differentiating infection from aseptic loosening or are limited by artifacts due to the prosthesis itself [2], [7]–[9]. Although the isolation of organisms and the histological analysis of intraoperative samples seem to be the best ways to confirm the diagnosis of PJI [10], [11], preoperative diagnostic tests can allow earlier diagnosis of PJI.

Nuclear medicine procedures can provide more specific physiological information about PJI. The technetium scan is performed first to show all areas of high metabolic activity. Combining technetium-99 m bone scans with conventional radiographs may slightly increase the sensitivity of diagnosis compared with the review of radiographs alone [12]. Radioisotopes targeting the white blood cells that are invariably present during infection can also be helpful in certain cases [13]. Anti-granulocyte scintigraphy using monoclonal antibodies or antibody fragments directly targets leukocyte antigens or receptors in vivo and allows the exploitation of the high granulocyte concentrations in the inflamed tissue surrounding the prosthesis after total joint arthroplasty. The anti-granulocyte scintigraphy scans help to distinguish true infection from uninflamed areas of high metabolic activity. The agents most commonly used to image prosthesis infections are immunoglobulin G (IgG) antibodies against normal cross-reactive antigen-95 (anti-NCA-95, 99 mTc-BW250/183) and the Fab fragment of the IgG antibody directed against the glycoprotein cross-reactive antigen-90 (anti-NCA-90, 99 mTc-sulesomab, LeukoScan®). Technetium-99 m-IgG scintigraphy is a highly sensitive technique for the recognition of infection around hip and knee prostheses; unfortunately, this method has a low specificity [14]. In particular, 99 mTc-sulesomab has been increasingly used for the diagnosis of PJI after arthroplasty, with a variety of reported outcomes. Although several studies have evaluated the accuracy of these antibodies for the diagnosis of PJI, the small sample size limited these studies. Therefore, studies have not provided conclusive information about the diagnostic accuracy of the anti-granulocyte scintigraphy with 99 mTc-labeled monoclonal antibodies. Additionally, heterogeneity in the primary diagnostic studies complicates the interpretation of these results. To provide more information regarding the use of anti-granulocyte scintigraphy with 99 mTc-monoclonal antibodies for the diagnosis of PJI after total joint arthroplasty, this meta-analysis summarizes the available evidence for its diagnostic accuracy.

## Materials and Methods

### Search Strategy

We performed a systematic search of the Medline, Embase, ScienceDirect, and OVID databases to identify epidemiological studies published through January 2013 that were related to the diagnostic test accuracy of anti-granulocyte scintigraphy with 99 mTc-monoclonal antibodies in the identification of PJI after total joint arthroplasty. Relevant prospective or retrospective cohort or case-control studies were included in the meta-analysis. The following search terms were adopted for the search of each database: anti-granulocyte scintigraphy, leukocyte scintigraphy, monoclonal antibody, sulesomab, BW 250/183, prosthesis infection, and total joint arthroplasty. The controlled vocabulary search terms for different databases are not identical. Therefore, search strategies need to be customized for each database. Only English-language studies were included in the meta-analysis. Furthermore, the reference lists of all full-text papers were examined to identify any studies which were initially omitted.

### Eligible Criteria

#### Inclusion criteria

Studies were considered eligible for inclusion if they met the following criteria:

**Study design.** Observational studies (cohort or case-control studies).

**Population.** Patients with PJI, without PJI, or suspected PJI after total joint arthroplasty (hip, knee, shoulder, or elbow).

**Diagnostic test.** Anti-granulocyte scintigraphy with monoclonal antibodies or antibody fragments labeled with technetium 99 m.

**Reference test.** The following reference tests were considered eligible: bacteriological culture, radiologic examination (X-ray, CT, MRI), clinical follow-up examination, CRP, ESR, peripheral leukocyte count, histological examination, cell cultures, etc.

#### Exclusion criteria

Studies were excluded from the meta-analysis for the following reasons: (1) Duplicate publication; (2) No human studies; (3) Necessary data could not be obtained.

### Study Selection

Two reviewers independently screened the titles and abstracts for studies which met the eligibility criteria. Subsequently, the full text of the studies that potentially met the inclusion criteria were read, and the literature was reviewed to determine the final inclusion. We resolved disagreements by reaching a consensus through discussion.

### Data Abstraction

Two of the authors independently extracted specific data from each full-text report using a standard data extraction form. The data obtained from the studies included the title, authors, year of publication, study design, number of eligible patients, type of joint arthroplasty, type of monoclonal antibody or antibody fragment, time between the prosthesis implantation and the anti-granulocyte scintigraphy, definition of positivity, and reference test.

### Assessment of Methodological Quality

Although evaluating study quality can help explain the heterogeneity of study outcomes, there is no consensus regarding the best way to incorporate quality during analysis. The methodological quality of the included studies was independently assessed by two authors, using the Quality Assessment of Diagnostic Accuracy Studies (QUADAS) list, which consists of 20 items scored as “yes,” “no” or “unclear” [15]–[17]. The scoring criteria are available upon request. Any disagreements were resolved by discussion. A third author was the adjudicator when a consensus could not be reached. An experienced nuclear physician was consulted for the assessment of the test technology used (item 13). No summary quality scores or weights for the different quality items were applied, because the interpretation of summary scores can be problematic and potentially misleading [18], [19].

### Statistical Analysis

Standard methods recommended for meta-analysis of diagnostic accuracy were used. The true positive rate (TPR) and false positive rate (FPR) of each study were converted by constructing a 2×2 contingency table, and the patient numbers were used to calculate the overall diagnostic accuracy. The following indexes of test accuracy were computed for each study: sensitivity, specificity, positive likelihood ratio (PLR), negative likelihood ratio (NLR), and diagnostic odds ratio (DOR). The DOR is an indicator of diagnostic accuracy that combines the sensitivity and specificity data into a number. The DOR describes the odds of a positive test result in patients with PJI compared with the odds of a positive test result in patients without PJI [20]–[23]. The DOR value ranges from 0 to infinity, with higher values indicating higher accuracy levels [23]. Additionally, we summarized the joint distribution of true positive and true negative rates in a summary receiver operating characteristic (SROC) curve. The area under the curve (AUC) represents an analytical summary of the test performance and illustrates the trade-off between sensitivity and specificity. The Q* index is the highest point in the SROC curve which intersects the antidiagonal where the sensitivity and specificity are equal and represents a summary of the test performance. The AUC and Q* index values range between 0 and 1, and higher values indicate better test performance than lower values [24], [25]. The interstudy heterogeneity was assessed using the Cochrane Q statistic. Because these studies were clinically heterogeneous, the pooled sensitivity, specificity, PLR, NLR, and DOR were calculated with a random-effects model with corresponding 95% confidence intervals (CIs). We also calculated the Spearman correlation coefficients. A strongly positive rank-correlation coefficient and a *p* value of <0.05 are indicative of a significant threshold effect. As publication bias is of concern for the meta-analyses of diagnostic studies, we tested for the potential presence of this bias using Deeks’ funnel plots [26]. All analyses were performed using 2 statistical software programs, Stata, version 12.0 (Stata Corporation, College Station, TX, USA) and Meta-Disc 1.4 for Windows (XI Cochrane Colloquium, Barcelona, Spain). All statistical tests were two-sided, and significance was set at p<0.05.

## Results

### Search Results

**Figure 1:**
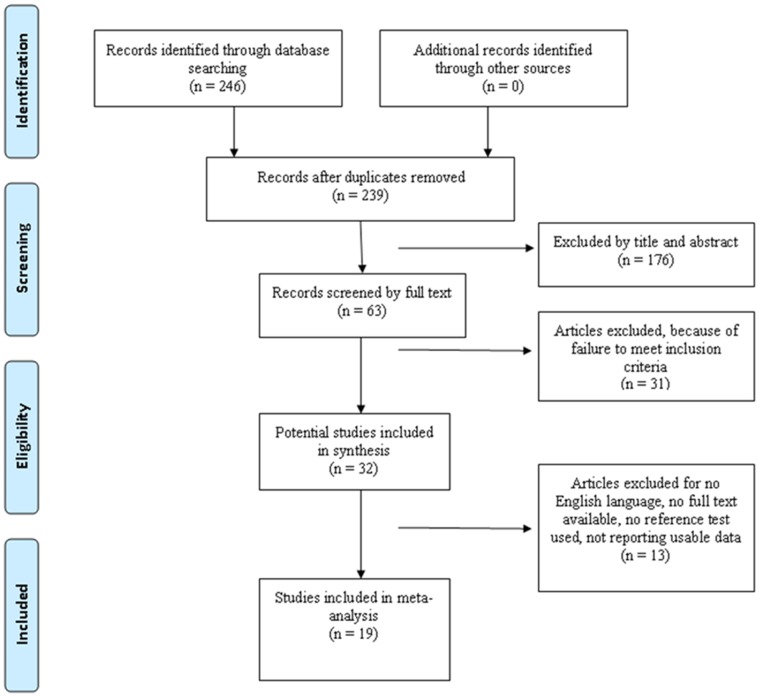
The study selection and inclusion process.

A total of 246 titles and abstracts were preliminarily reviewed, of which 19 studies [4], [27]–[44] eventually satisfied the eligibility criteria. Figure 1 shows a flow diagram of the selection process.

### Demographic Characteristics

Table 1 presents the characteristics of the included studies. These articles were published between 1992 and 2012 and included 17 cohorts (702 patients) and 2 case-control studies (53 patients). The individual studies concerned hip, knee, shoulder and elbow prostheses. The sample size ranged from 8 to 81 patients having undergone total joint arthroplasty. Of the included studies, 8 were conducted in Germany, 3 in the UK, 1 in Switzerland, 1 in Spain, 1 in Portugal, 1 in Italy, 1 in Greece, 1 in France, 1 in Finland, and 1 in Denmark. For anti-granulocyte scintigraphy after the administration of varying doses of 99 mTC, 15 of the studies used the monoclonal antibody sulesomab, while 4 of the studies used BW 250/183. Four of the included studies used semiquantitative criteria to interpret anti-granulocyte scintigraphy scans on the basis of the increase in the activity quotient during the late phase of examination versus that during the early phase. All of the following reference standards were used for the PJI diagnosis of each patient: bacteriological culture, histopathological examination, microbiologic or laboratory examination, clinical follow-up examination, and radiologic examination (X-ray, CT, MRI, and indium white blood cell scintigraphy).

**Table 1:** Characteristics of included studies.

N	Study	year	Country	Studydesign	Number ofprostheses	Site ofarthroplasty	Age	Male	Antibodytype	Dose	Scanningtime	Numberof readers	Interpretation	DiagnosisStandard
1	Sciuk et al. [41]	1992	Germany	Prospective cohort	43	H,K	61	47%	BW 250/184	400–500	4 and 24	2	Semiquantitative	C,H,FU
2	Boubaker et al. [4]	1995	Switzerland	Prospective cohort	75	H	73	60%	BW 250/183	750	1,6 and 24	2	Semiquantitative	C,H,FU
3	Devillers et al. [40]	2000	France	Prospective cohort	8	H,K	71	63%	Sulesomab	900	1 and 4–6	3	Qualitative	C,H,R,FU
4	Ivancevic et al. [38]	2002	Germany	Retrospective cohort	26	H,K	62	NR	Sulesomab	<1000	1,5 and 24	3	Semiquantitative	C,H
5	Ryan et al. [39]	2002	UK	Case-control	23	K	NR	NR	Sulesomab	750	2 and 6	1	Qualitative	C,M,R,FU
6	Larikka et al. [33]	2002	Finland	Case-control	30	H	65	37%	Sulesomab	370	3	2	Qualitative	C,M,FU
7	Gratz et al. [37]	2003	Germany	Retrospective cohort	20	H,K	58	NR	Sulesomab	1110	1,4 and 24	NR	NR	H,FU
8	Klett et al. [44]	2003	Germany	Retrospective cohort	26	K	69	38%	BW 250/184	600–800	4–6 and 23–25	NR	Qualitative	H
9	von Rothenburget al. [34]	2004	Germany	Retrospective cohort	38	H,K	71	24%	Sulesomab	555–925	2 and 4–6	2	Qualitative	C,H,M
10	Rubello et al. [35]	2004	Italy	Retrospective cohort	78	H,K,F	NR	NR	Sulesomab	740	4 and 18–24	2	Qualitative	M,R,FU
11	Vicente et al. [36]	2004	Spain	Retrospective cohort	81	H,K	64	NR	Sulesomab	740	3–4 and 7–8	2	Qualitative	C,M,FU
12	Iyengar et al. [32]	2005	UK	Retrospective cohort	38	H,K,E,S	72	47%	Sulesomab	650	4–5	NR	Qualitative	C,H,M,R,FU
13	Simonsen et al. [30]	2007	Denmark	Retrospective cohort	76	H	73	46%	Sulesomab	628	1,3 and 22	2	Qualitative	C,H,M,FU
14	Pakos et al. [31]	2007	Greece	Retrospective cohort	19	H,K	67	NR	Sulesomab	740	2	2	Qualitative	C,H,M,FU
15	Rubello et al. [43]	2008	UK	Prospective cohort	78	K	58	35%	Sulesomab	740	4 and 20–24	2	Semiquantitative	R
16	Gratz et al. [42]	2009	Germany	Retrospective cohort	26	H,K	NR	35%	Sulesomab	1000–15004 and 24		2	Qualitative	C,M,FU
17	Graute et al. [29]	2010	Germany	Retrospective cohort	31	H,K,S	68	42%	BW 250/184	727–877	4–6 and 23–25	2	Qualitative	C,H,M,FU
18	Sousa et al. [28]	2011	Portugal	Prospective cohort	19	H,K	NR	NR	Sulesomab	740	2 and 4	3	Qualitative	C,H,M,FU
19	Gratz et al. [27]	2012	Germany	Prospective cohort	20	K	68	NR	Sulesomab	740	4 and 24	2	Qualitative	C,M,FU

NR: not reported; C: bacteriological culture; M: microbiologic or laboratory examination; H: histological examination; R: radiological examination; H: hip; K: knee; E: elbow; S: shoulder.

### Assessment of Methodological Quality

**Figure 2:**
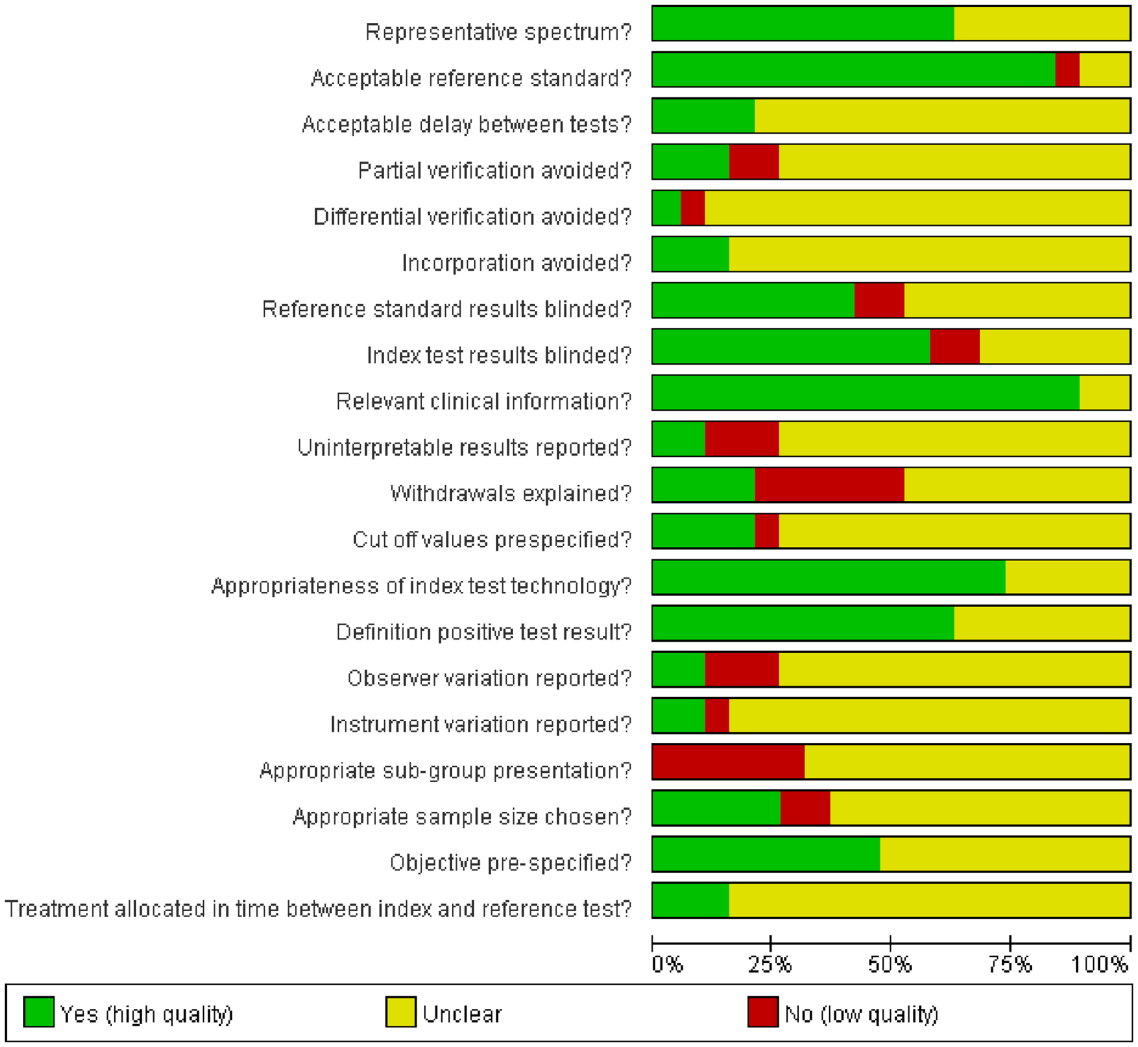
The assessment of methodological quality items shown as percentages across all included studies.

**Figure 3:**
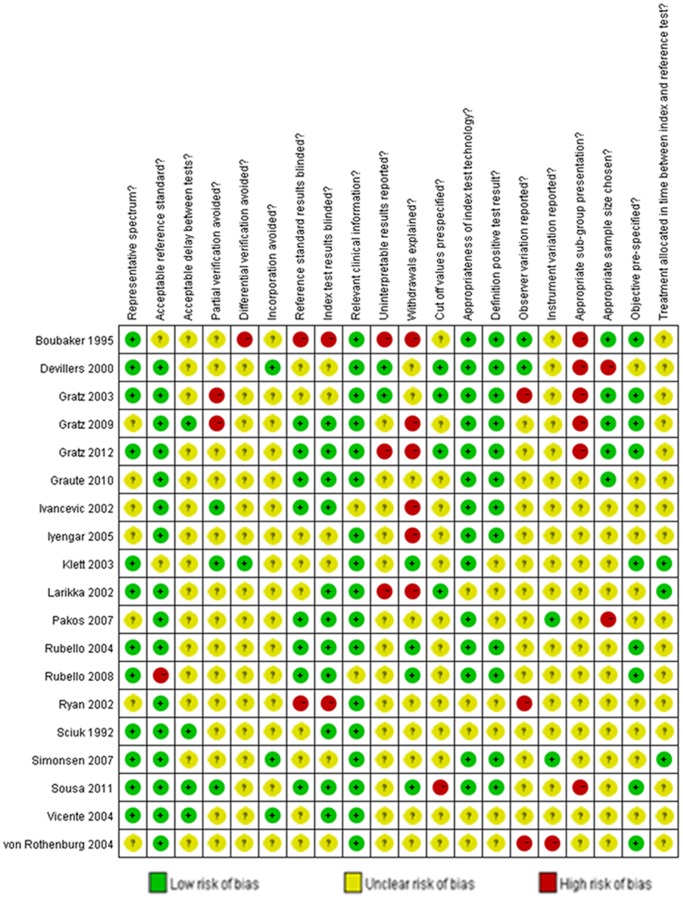
Methodological quality of each included study.

Judgments about each risk of bias item are shown as percentages across all of the included studies in Figure 2. The methodological quality of the included studies is presented in Figure 3. There was a large variation in methodological quality of included studies. Poor reporting of several quality items exerted an influence on the validity of the reported sensitivities and specificities and impeded the assessment of the risk of bias.

### Diagnostic Results of Included Studies

Table 2 provides detailed data regarding the sensitivity, specificity, and other diagnostic results.

**Table 2:** Summary of the diagnostic results of the included studies.

N	Study	Year	Sample size	TP	FP	FN	TN	Sensitivity	Specificity
1	Sciuk et al. [41]	1992	43	16	4	2	21	0.89	0.84
2	Boubaker et al. [4]	1995	75	8	16	4	47	0.67	0.75
3	Devillers et al. [40]	2000	8	3	1	0	4	1.00	0.80
4	Ivancevic et al. [38]	2002	26	10	5	0	11	1.00	0.69
5	Ryan et al. [39]	2002	23	4	2	2	15	0.67	0.88
6	Larikka et al. [33]	2002	30	5	0	3	22	0.63	1.00
7	Gratz et al. [37]	2003	20	5	1	3	11	0.63	0.92
8	Klett et al. [44]	2003	28	13	3	0	12	1.00	0.80
9	von Rothenburg et al. [34]	2004	38	14	8	1	15	0.93	0.65
10	Rubello et al. [35]	2004	78	48	5	9	16	0.84	0.76
11	Vicente et al. [36]	2004	81	12	7	3	59	0.80	0.89
12	Iyengar et al. [32]	2005	38	10	5	1	22	0.91	0.81
13	Simonsen et al. [30]	2007	76	22	3	5	46	0.81	0.94
14	Pakos et al. [31]	2007	19	9	1	3	6	0.75	0.86
15	Rubello et al. [43]	2008	78	38	8	3	29	0.93	0.78
16	Gratz et al. [42]	2009	26	12	3	9	2	0.57	0.40
17	Graute et al. [29]	2010	31	6	9	3	13	0.67	0.59
18	Sousa et al. [28]	2011	19	4	12	0	3	1.00	0.20
19	Gratz et al. [27]	2012	20	14	1	0	5	1.00	0.83

TP: true positive; FP: false positive; FN: false negative; TN: true negative.

### The SROC

**Figure 4:**
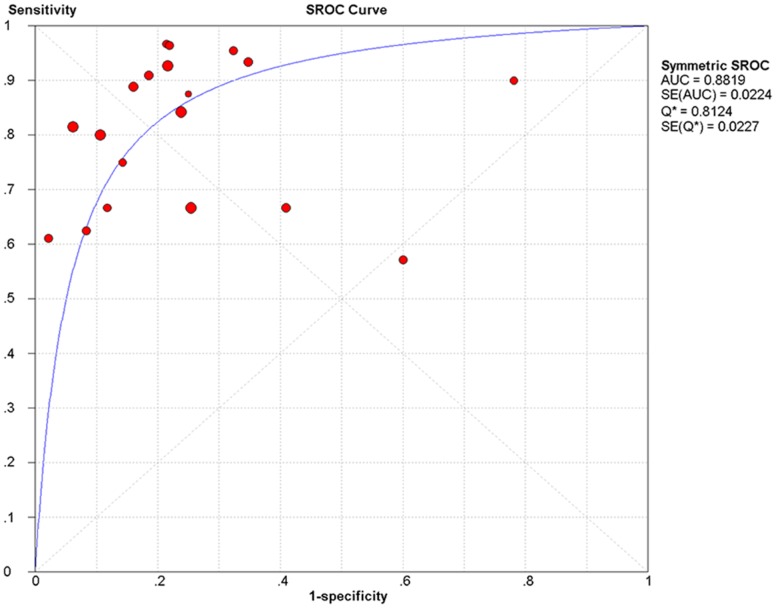
Summary receiver operating characteristic (SROC) curve for anti-granulocyte scintigraphy with 99mTc-monoclonal antibodies in the diagnosis of periprosthetic joint infections (PJI) in the 19 included studies. Solid circles represent each study included in the meta-analysis. The size of each study is indicated by the size of the solid circle. The regression SROC curve summarizes the overall diagnostic accuracy. AUC (area under the curve)  =  0.88, Q*  =  0.81.

The corresponding SROC (Figure 4) shows an AUC of 0.88 with standard error = 0.02, and the pooled diagnostic accuracy (Q*) was 0.81 with standard error = 0.02, indicating high overall accuracy of anti-granulocyte scintigraphy with 99 mTc-monoclonal antibodies for the diagnosis of PJI after total joint arthroplasty. The Spearman rank correlation coefficient was 0.24 (p = 0.32), confirming that the variability across these studies could not be explained by differences in the diagnostic threshold.

### The Pooled DOR

**Figure 5:**
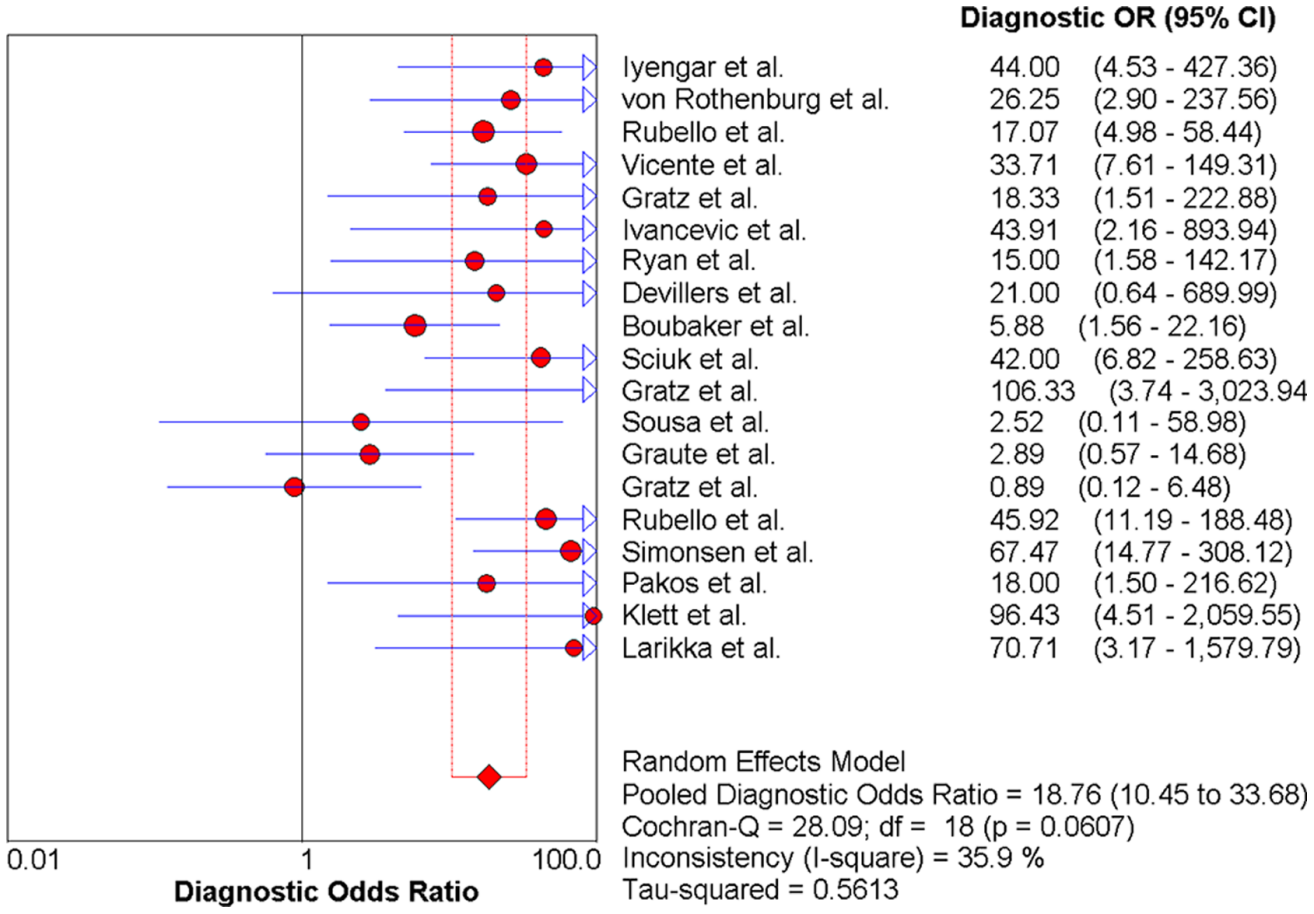
Forest plot for the diagnostic odds ratio (DOR) of anti-granulocyte scintigraphy with 99mTc-monoclonal antibodies to diagnose periprosthetic joint infections (PJI). DOR (diagnostic odds ratio)  =  18.76 (95% CI, 10.45-33.68).

Significant heterogeneity among the studies was not detected (Cochran Q statistic = 28.09; P = 0.06). A Forest plot for the DOR of anti-granulocyte scintigraphy with 99 mTc-monoclonal antibodies for the diagnosis of PJI was 18.76 with a corresponding 95% CI of 10.45–33.68, as shown in Figure 5.

### The Pooled Sensitivity and Specificity

**Figure 6:**
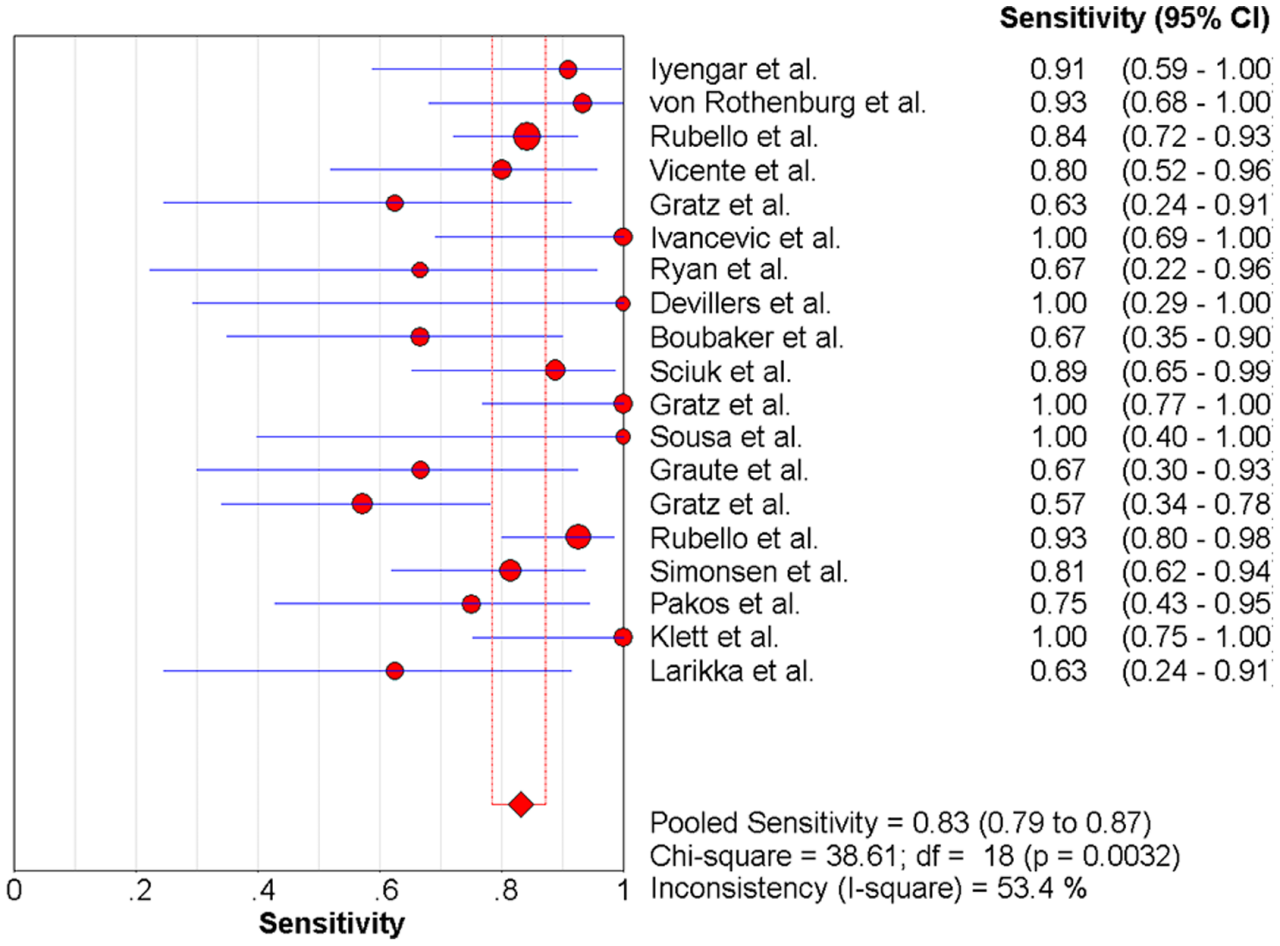
Forest plot for the sensitivity of anti-granulocyte scintigraphy with 99mTc-monoclonal antibodies to diagnose periprosthetic joint infections (PJI). Sensitivity  =  0.83 (95% CI, 0.79-0.87).

**Figure 7:**
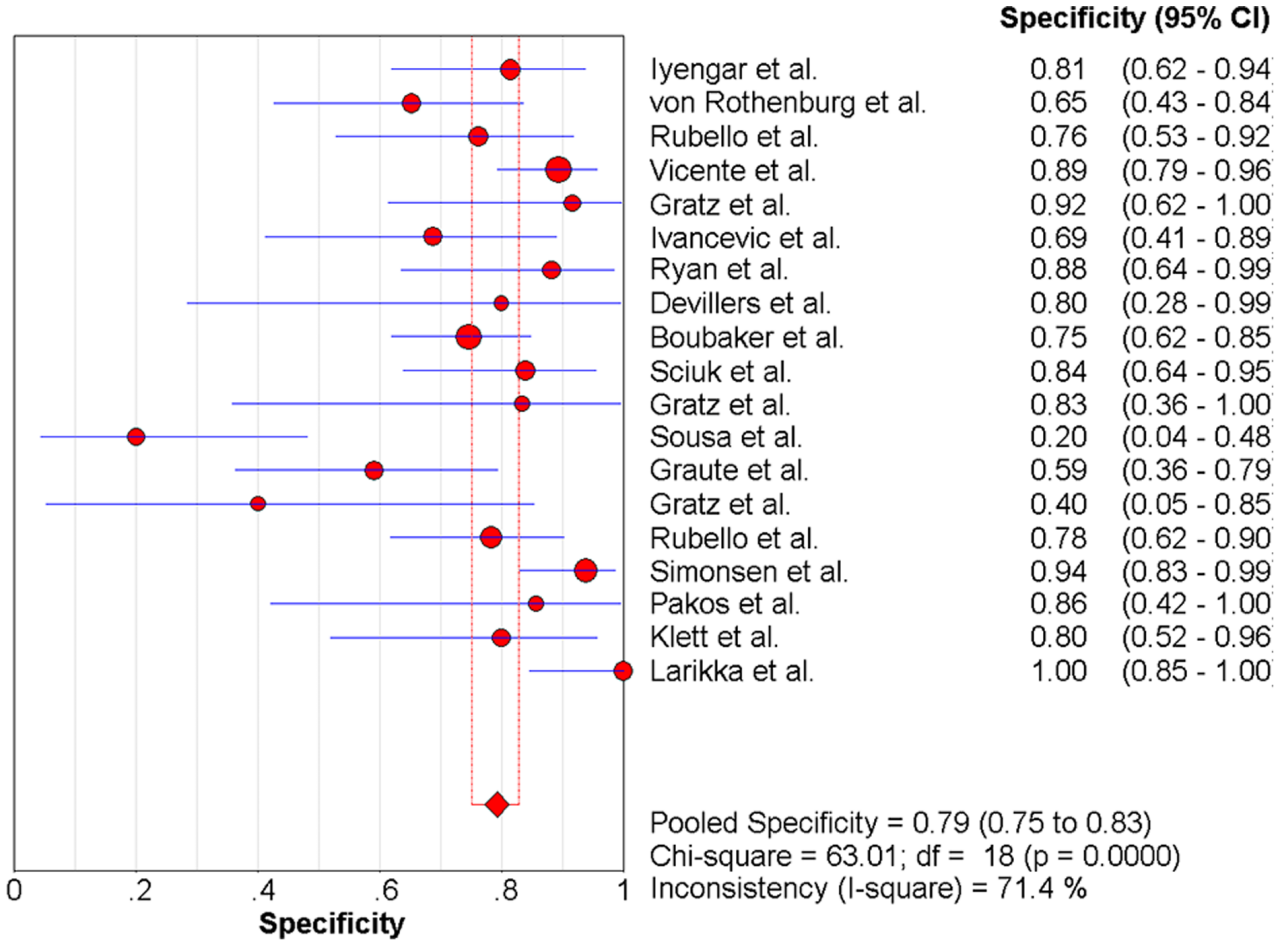
Forest plot for the specificity of anti-granulocyte scintigraphy with 99mTc-monoclonal antibodies to diagnose periprosthetic joint infections (PJI). Specificity  =  0.79 (95%CI, 0.75-0.83).

Significant heterogeneity among the studies was detected (sensitivity: chi-square = 38.61, P = 0.003, Figure 6; specificity: chi-square = 63.01, p<0.0001, Figure 7). The sensitivity ranged from 57% to 100% (pooled, 83%; 95% CI, 79–87%), whereas specificity ranged from 20% to 100% (pooled, 79%; 95% CI, 75–83%).

### The Pooled Likelihood Ratio (PLR and NLR)

**Figure 8:**
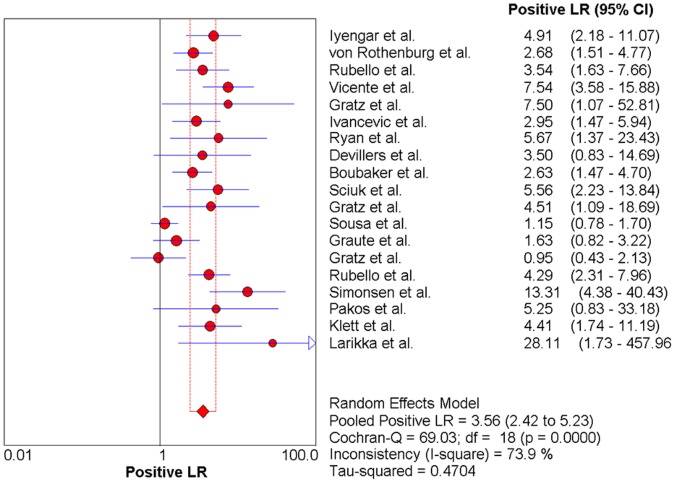
Forest plot for the positive likelihood ratio (PLR) of anti-granulocyte scintigraphy with 99mTc-monoclonal antibodies to diagnose periprosthetic joint infections (PJI). PLR (positive likelihood ratio)  =  3.56 (95% CI, 2.42-5.23).

**Figure 9:**
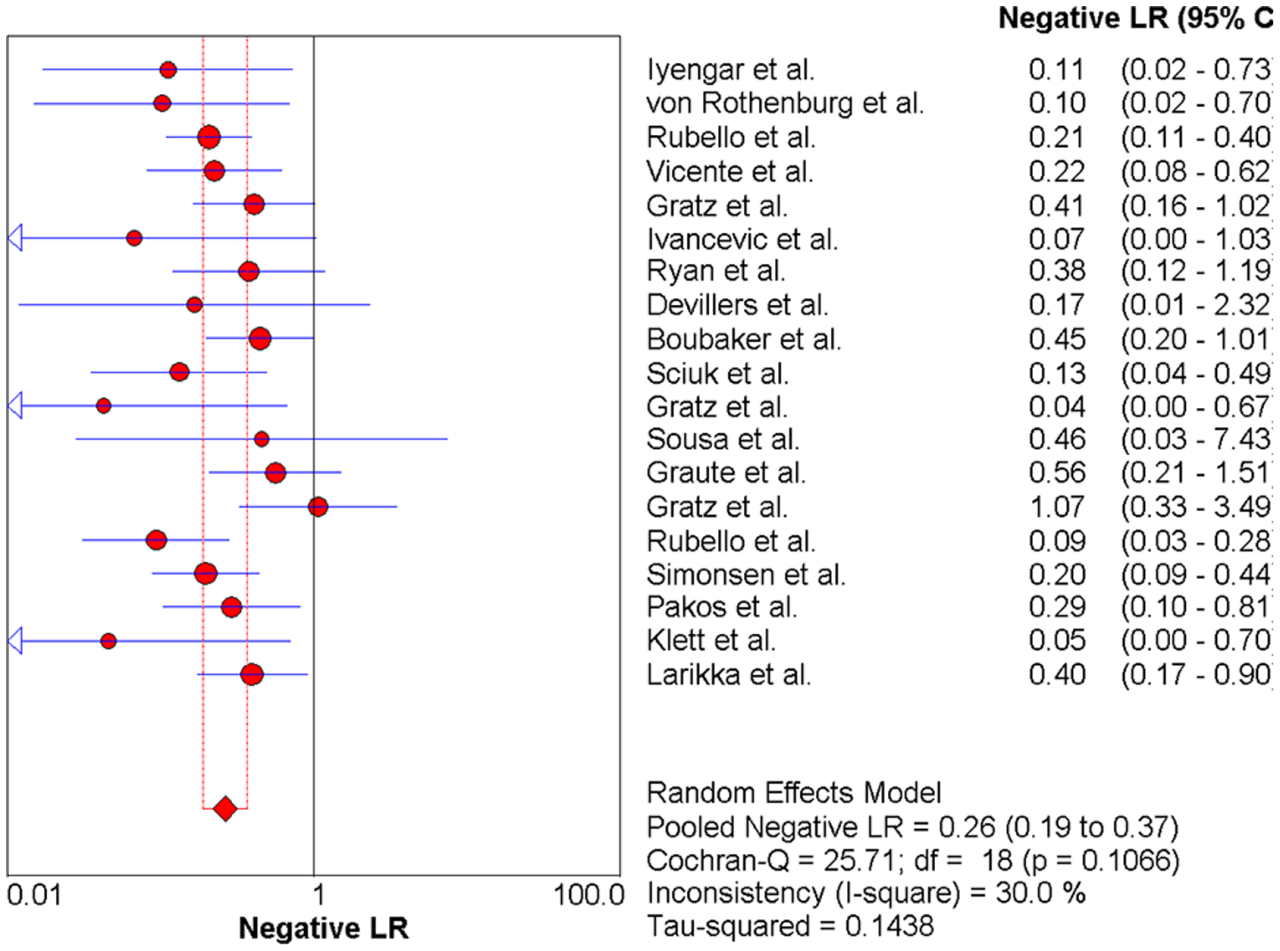
Forest plot for the negative likelihood ratio (NLR) of anti-granulocyte scintigraphy with 99mTc-monoclonal antibodies to diagnose periprosthetic joint infections (PJI). NLR (negative likelihood ratio)  =  0.26 (95% CI, 0.19-0.37).

Significant heterogeneity among the studies was also detected in the PLR (Cochran Q statistic = 69.03, p<0.001, Figure 8). However, no significant heterogeneity was found in the NLR (Cochran Q statistic = 25.71, p = 0.11, Figure 9). The pooled PLR was 3.56 (95% CI, 2.42–5.23), and the pooled NLR was 0.26 (95% CI, 0.19–0.37).

### Publication Bias

**Figure 10:**
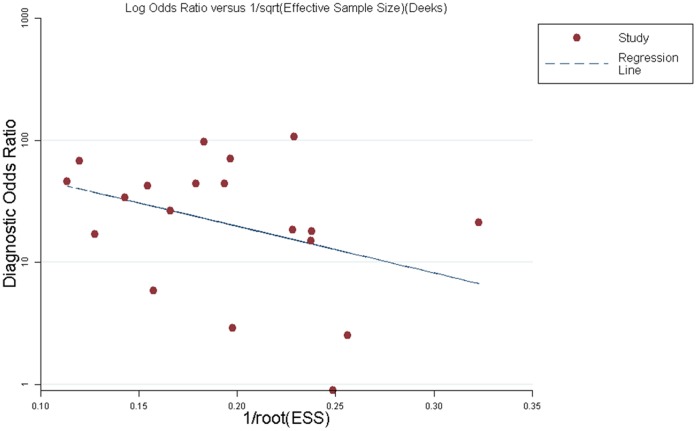
Funnel plot for the assessment of potential publication bias of the 19 included studies. The funnel graph plots the log of the diagnostic odds ratio (DOR) against the standard error of the log of the DOR (an indicator of sample size). Solid circles represent each study in the meta-analysis. The line indicates the regression line. There was no potential publication bias.

Although the funnel plots for publication bias showed some asymmetry due to the limited number of included studies (Figure 10), the result of Deeks’ test was non-significant (p = 0.11), indicating that there was no potential publication bias.

## Discussion

Diagnosing PJI after total joint arthroplasty is a crucial and complex task. At present, no single laboratory test has perfect sensitivity and specificity for the diagnosis of PJI [10], and therefore the surgeon is forced to make a decision based on the collective interpretation of different test modalities. Although there was a published new definition for PJI proposed by Musculoskeletal Infection Society in 2011, numerous other tests was being evaluated [45]. The evaluation of the white blood cell counts, differential blood cell counts and erythrocyte sedimentation rates (ESR) has demonstrated that these diagnostic methods lack both sensitivity and specificity in determining the presence of potential PJI after total joint arthroplasty [5], [46]–[48]. Radiography cannot distinguish between early mechanical loosening and low-grade sepsis [49]. Histologic examinations have been used to confirm the diagnosis of infection but have lacked the necessary sensitivity to rule out infection as a cause of prosthetic joint loosening [50]. Intraoperative cultures may be negative for some patients with clinically proven PJI. The culture of aspirated material can confirm the diagnosis of infection, but given the low sensitivity of this test, a negative result does not rule out the presence of infection [14]. Thus, the diagnosis of PJI after total joint arthroplasty remains a challenge.

Nuclear imaging studies have been the subject of numerous investigations for the diagnosis of PJI. Conventional bone scintigraphy is a highly sensitive method for detecting bone infection, but lacks the specificity needed to differentiate between infection, heterotrophic ossification, fracture, neoplasms and arthritis [51]. Although gallium scans are also used, gallium can accumulate in noninfected areas of increased bone turnover [52]. Scintigraphy with In-111-oxine-labeled autologous leukocytes is generally used only in specialized centers because of the increased risk of infection, the extensive time involved, the increased exposure to radiation, and the suboptimal imaging quality [53]. Thus, the focus of interest has been on Tc-99 m-labeled monoclonal antibodies, which are simple to use. Anti-granulocyte antibodies (Fab fragment of the IgG antibody against the glycoprotein cross-reactive antigen-90 and IgG antibody against normal cross-reactive antigen-95) are not only found in the infected tissue surrounding prosthesis due to increased granulocyte concentrations but also in the bone marrow as a result of phagocytosis by the reticuloendothelial cells [54]. Anti-granulocyte scintigraphy with monoclonal antibodies has increasingly been used for the diagnostic evaluation of suspected PJI after total joint replacement. However, the limited sample size of the included studies limited the statistical power of the evaluation of anti-granulocyte scintigraphy with monoclonal antibodies in the identification of PJI after total joint arthroplasty. Therefore, it was imperative to pool the results of individual studies to evaluate the diagnostic value of this method via meta-analysis.

In all studies included in our meta-analysis, QUADAS was applied to ensure that most of the selected articles were moderate-quality. The methodological quality assessment identified a number of limitations to the current evidence base. The quality of the included studies may influence the reliability of the results. Significant heterogeneity among the included studies was confirmed with the Cochrane Q statistic. One of the most important sources of heterogeneity was the lack of a gold standard test. This resulted in a large variation of reference tests. Due to the absence of a gold standard test, misclassification bias may affect the estimates of diagnostic accuracy. The comparison of the present method against different reference tests could lead to an underestimation of the diagnostic accuracy. However, the combination of several reference tests in the individual studies may mitigate this effect. In the present study, original studies were included if they used bacteriological culture, histopathological examination, laboratory examination, clinical follow-up examination, or radiological examination as a reference test. Nevertheless, it was impossible to perform sensitivity analyses or stratification according to the type of reference tests, because most eligible studies did not provide separate results based on the reference tests. The isolation of organisms and histological analysis of intraoperative samples is often regarded as the best reference test to definitively confirm the diagnosis of PJI [10], but these tests are subject to partial verification, as only patients with strongly suspected underlying causes are generally subjected to surgery. Verification bias might result in a lower specificity and higher sensitivity [55], while this bias has also been found to increase both specificity and sensitivity [56].

Furthermore, some degree of heterogeneity was also induced by the variability of the patients included. These individuals had different types of arthroplasty and different sites of total join arthroplasty. In addition, different types of antibodies, study designs, doses of monoclonal antibodies or technetium 99 m-labeled antibody fragments, and different scanning times were all used. Although the random effects model incorporates heterogeneity, it is still possible that the pooled results of diagnostic accuracy of anti-granulocyte scintigraphy with 99 mTc-monoclonal antibodies are affected by the above factors. The summarized results were based on a limited number of studies of moderate quality with several unaddressed sources of heterogeneity, and the generalizability and validity of the results are therefore limited. Moreover, significant heterogeneity was detected in the sensitivity, specificity and PLR, but not in the DOR or NLR. This may have been caused by higher levels of variation in the sensitivity, specificity and PLR between individual studies. However, the inconsistency of the results may be related to all of the above sources of heterogeneity. Therefore, the underlying reason for the inconsistency of the results could not be absolutely determined based on the current evidence, due to the paucity or lack of reported data regarding these variables in the included studies. Accordingly, although the results of the meta-analysis should be considered appropriate, the methodological quality and clinical heterogeneity should also be considered when interpreting the findings.

This meta-analysis summarizes the evidence for the diagnostic accuracy of anti-granulocyte scintigraphy with monoclonal antibodies or technetium-99 m-labeled antibody fragments in patients with PJI after total joint arthroplasty. The pooled results showed a sensitivity of 0.83, specificity of 0.79, and AUC of 0.88, indicating a relative level of overall accuracy. The above sensitivity was lower than ESR and CRP which are the ubiquitous, inexpensive, low risk diagnostic tests, while these serum markers could be affected by conditions remote from the joint prostheses [57]. Thus, the anti-granulocyte scintigraphy with 99 mTc-labeled monoclonal antibodies could offer higher specificity than CRP and ESR. The present study showed that the pooled DOR was 18.76, which indicated a high level of overall accuracy. However, the SROC curve and the DOR are difficult to interpret and are therefore not used in clinical practice [58]. The PLR and NLR are more clinically meaningful indicators of diagnostic accuracy. High PLR and low NLR values indicate that a method is highly discriminating. Although there is no absolute threshold, a good diagnostic test may have a PLR>5 and an NLR<0.2 [59]. However, the PLR and NLR values of this study did not meet these cutoff values. In the present meta-analysis, a PLR value of 3.56 revealed that patients with PJI had approximately a 3.56-fold higher chance of testing positive than patients without PJI, and this was relatively high for clinical purposes. On the other hand, an NLR value of 0.26 demonstrated that a patient with PJI had a 26% chance of testing negative, and this method is therefore not sensitive enough to rule out PJI in the case of a negative test. These results suggest that a substantial proportion of patients might be incorrectly classified according to the anti-granulocyte scintigraphy with 99 mTc-labeled monoclonal antibodies. Based on the current pooled evidence, using isolated anti-granulocyte scintigraphy with 99 mTc-monoclonal antibodies will help to diagnose PJI, but may not fully replace other routine diagnostic methods such as CRP, ESR, bacteriologic culture and histologic examination, which have been used for the diagnosis of PJI. An accurate diagnosis of PJI often requires the use of combinations of tests and a strong clinical suspicion.

The present meta-analysis is useful to define the optimal spectrum of applications for this diagnostic technology. However, the importance of these findings should be interpreted based on the clinical consequences. The role of anti-granulocyte scintigraphy largely depends on the suspected underlying pathology as well as the setting and patient characteristics. Sousa et al. [28] reported that this new nuclear medicine modality may provide an alternative to autologous-labeled leukocytes. Graute et al. [29] reported that this diagnostic method seems suited for those patients requiring surgical therapy. Gratz et al. [42] reported that the diagnostic technology was highly sensitive and specific for diagnostic imaging of infection in patients after total knee arthroplasty. Conservatively, anti-granulocyte scintigraphy with 99 mTc-monoclonal antibodies seems to be a complementary diagnostic method to traditional diagnostic tests such as histological biopsy and bacteriological culture. However, the consistency in requesting or performing the test is dependent on the preference of the orthopedist and the availability of an experienced radiologist [50]. Although anti-granulocyte scintigraphy with monoclonal antibodies or technetium-99 m-labeled antibody fragments is not 100% accurate in the diagnosis of PJI, it is one of several diagnostic methods that help an orthopedist to make a decision regarding the infection status of a patient.

The primary limitations of this systematic review include the following: (1) Wide confidence intervals were induced by the small number of patients in the eligible studies. Collecting large sample sizes of patients with suspected infections was difficult. The statistical efficacy could be improved by including more studies. (2) The reliability of the pooled estimates is dependent on the methodological quality of the included studies. Although eligible studies meet many of the a priori quality metrics, weaknesses remain. (3) Other factors in the included studies, such as age, type of prostheses, patient spectrum, and expertise with this scintigraphy technology could not be addressed. (4) The indications for an imaging diagnostic technology and the recruitment of patients are very difficult to study in randomized control trials. In terms of reporting the diagnostic methods for PJI, future studies should provide more detailed inclusion and exclusion criteria, as well as baseline characteristics of the participants, including prosthesis age, patient age, joint disease, and antecedent use of antibiotics. (5) Moreover, given the various reference diagnostic tests used, methodological shortcomings of individual studies are inevitable. Thus, high-quality, well-designed studies are required to evaluate the sensitivity and specificity of the anti-granulocyte scintigraphy with 99 mTc-monoclonal antibodies, while controlling for as many variables as possible.

## Conclusions

The present meta-analysis demonstrated that anti-granulocyte scintigraphy with monoclonal antibodies or technetium-99 m-labeled antibody fragments has a role in the diagnosis of PJI after total joint arthroplasty. The results of this diagnostic method should be interpreted in parallel with clinical findings and other conventional tests. We believe that evaluation of the present diagnostic method will provide evidence to aid orthopedists in diagnosing PJI in patients after total joint arthroplasty. Due to the limitations of the present meta-analysis, additional high-quality original studies are required to confirm the predictive value.

## Supporting Information

Table S1 **Preferred reporting items for systematic reviews and meta-analyses (PRISMA) 2009 Checklist.**
(DOC)
